# Frameshift Mutations (Deletion at Codon 1309 and Codon 849) in the APC Gene in Iranian FAP Patients: a Case Series and Review of the Literature

**Published:** 2014

**Authors:** Seyed Mohammad Hossein Kashfi, Faeghe Behboudi Farahbakhsh, Mina Golmohammadi, Ehsan Nazemalhosseini Mojarad, Pedram Azimzadeh, Hamid Asadzadeh Aghdaie

**Affiliations:** 1 Gastroenterology and Liver Diseases Research Center, Shahid Beheshti University of Medical Sciences, Tehran, Iran.; 2 Basic and Molecular Epidemiology of Gastrointestinal Disorders Research Center, Shahid Beheshti University of Medical Sciences, Tehran, Iran.

**Keywords:** Adenomatous polyposis coli, colorectal cancer, frameshift mutation

## Abstract

Familial adenomatous polyposis (FAP) is responsible for <1% of colorectal cancer (CRC) cases and is inherited an autosomal dominant trait. Patients generally present hundreds to thousands of adenomas and develop colorectal cancer by age 35- 40 if left untreated. Here we report four patients with germline frameshift mutation (small deletion) at exon 15 of adenomatous polyposis coli (*APC*) tumor suppressor gene. Peripheral blood samples were collected from patients and Exon 15 of the APC gene was studied by direct sequencing after genomic DNA extraction. Four frameshift mutations were detected. Two patients had 5 bp deletion, c.3927_3931delAAAGA and two siblings presented deletion at codon 849 (c.2547_2548delTA p.Asp849fsX62). This study was the first report of genetic screening in Iranian FAP patients. In contrast to other studies we revealed that one patient with mutation at codon 1309 had an attenuated phenotype.

Colorectal cancer is classified into two distinct groups: Sporadic and hereditary forms. Familial adenomatous polyposis (FAP) is an inherited autosomal dominant disease which is responsible for <1% of colorectal cancer (CRC) cases (1). Patients usually develop hundreds to thousands of adenomatous polyps in the colon and rectum and the incidence is equal in both genders (1-2). Approximately 15– 20% are considered as ‘‘de novo’’ cases without any family history of the disease (3). *FAP patients carry germline mutation at Adenomatous polyposis coli* (*APC*) *tumor suppressor gene which is located at the short arm of* chromosome 5 (5q21-22) and spans a region of 108,353bp (NC_000005) encoding a protein weighting 310 kDa (4). APC is involved in the different types of functions including cytoskeleton integrity, cellular adhesion and wingless/wnt signaling (5-6). This multifunctional protein is highly expressed in epithelial cells (7). The APC role in wnt signal transduction is vital (8). The wild type of APC negatively regulates β-catenin while mutant alleles result in β-catenin increasing in cytoplasm, inducing the transformation of colonic epithelial cells (9). Most of the APC mutations result in truncated proteins due to nonsense or frameshift mutations. One of the most common mutation sites in APC gene is codon 1309 (5 bp deletion, c.3927_3931delAAAGA). Severe Polyposis and early onset of colorectal cancer are two important features of patients with mutation at this codon (10-11). Beside polyposis which is common in patients with FAP, upper gastrointestinal manifestations are detected in 30- 100% of cases (12-13). In this study, four cases with germline frameshift mutation (small deletion) at exon 15 of APC tumor suppressor gene are presented and the correlation between phenotype and location of codons were evaluated in two cases.

**Case 1**

A 22-year-old female referred to Research Center for Gastroenterology and Liver Diseases (RCGLD) for her extreme abdominal pain and diarrhea which lasted for 3 months, a year ago. Patient underwent colonoscopy and profuse polyposis was detected throughout the colon. Pedigree was constructed and is shown in Fig. 1. The patient’s mother was diagnosed with polyposis and colorectal carcinoma when she was 53 years and died at age 55. Her brother also was diagnosed with FAP at age 16. As it is shown, the strong family history of colon cancer and FAP was present in the first degree the patient’s relatives. Colonoscopy report revealed numerous>100 small and microscopic adenomatous polyps (tubular adenomas) less than 0.1- 0.4 cm in diameters in cecum appendix and a rim of terminal ileum. Those were mostly mild and few with moderate dysplasia. Microscopic tubular adenoma was also detected in distal surgical margin. In abdominal wall mass, abdominal fibromatosis was confirmed (desmoid tumor). Total proctocolectomy with ileal pouch-anal anastomosis and protective ileostomy was performed in this case. Since the patient was diagnosed with FAP, we examined the mutation status in exon 15 of the APC gene. Mutation analysis was performed using polymerase chain reaction (PCR) and direct sequencing revealed a frameshift mutation with small deletion of two nucleotides (TA) at codon 849 (c.2547_2548delTA p.Asp849fsX62) of exon 15 (Fig. 2).

**Case 2**

A 17-year-old teenage boy referred to RCGLD because of abdominal pain and constipation. The patient underwent colonoscopy and several 5 mm to 10 mm polyps were observed in rectum (Fig. 3).

Polypectomy was performed. Pathology report revealed tubular adenomas with low grade dysplasia. As it is shown in his pedigree (Fig. 4), the patient’s brother was diagnosed with FAP and his father was diagnosed with colorectal cancer.

**Fig. 1:**
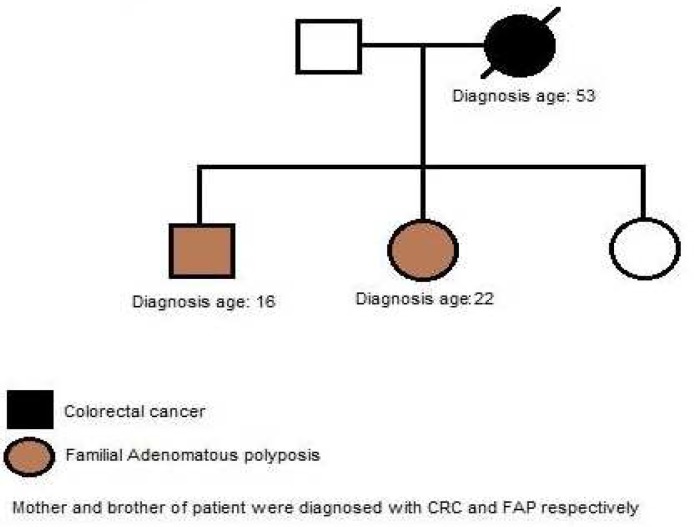
Mother and brother of patient were diagnosed with CRC and FAP respectively. Her mother died from CRC at age 55

**Fig. 2:**
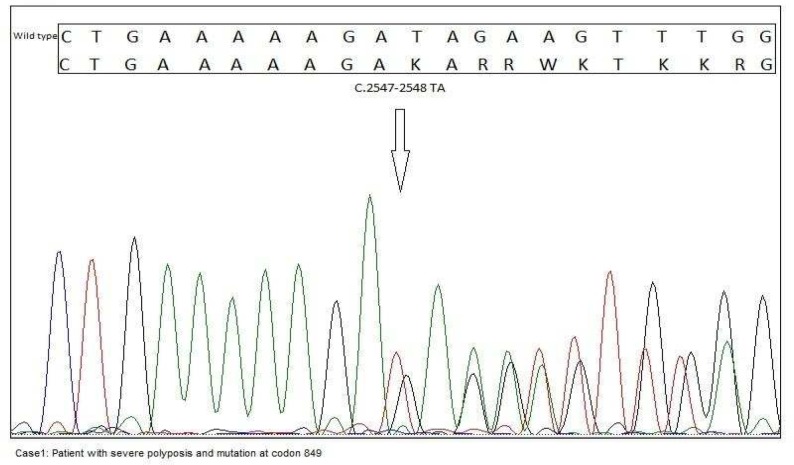
Sequencing revealed a frameshift mutation with small deletion of two nucleotides (TA) at codon 849 (c.2547_2548delTA p.Asp849fsX62) at exon 15 of APC

**Fig. 3:**
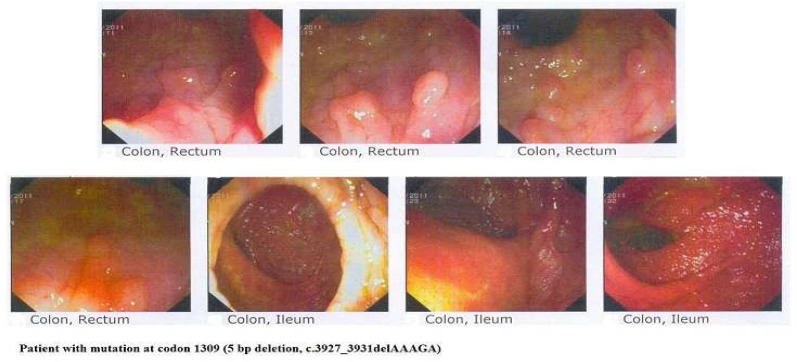
colonoscopy revealed multiple polyps in patient with mutation at codon 1309 (5 bp deletion, c.3927_3931delAAAGA

**Fig. 4:**
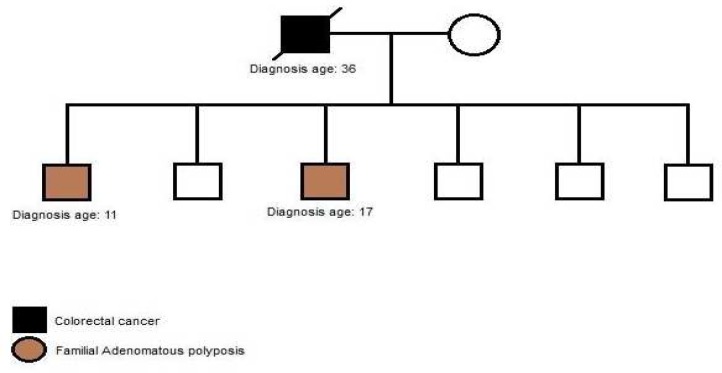
Patient diagnosed with FAP at age 17. Father of the patient died from CRC at age 39

After 1 year, the patient underwent colonoscopy and several 1 mm and 2 mm tubular adenomas polyps were detected in rectum. In terminal ileum one 15 mm obstructing tumor was seen. Terminal ileum biopsy showed mild active ileitis with prominent lymphoid follicular hyperplasia with no dysplasia. The patient underwent endoscopy because of his recurrent abdominal pain. In endoscopy procedure one 5.5 mm polyp in antrum and two sessile polyps 5.5 mm in fundus were observed and biopsies were taken. Antrum and fundus polyps consisted of hyperplastic polyps and no dysplasia or malignancies were detected. Antrum and fundus mucosa biopsy also showed moderate chronic gastritis and the patient was positive for* H.pylori*. The section of duodenum also showed an adenomatous polyp with low grade dysplasia. Screening of the APC gene in this case revealed mutation at codon 1309 (c.3927 _3931del-AAAGA p.Glu1309fs) (Fig. 5)

**Case 3**

A-16-year-old male referred to RCGLD for his abdominal pain and rectal bleeding. Colonos-copy report showed numerous polyps in the rectum and colon. His sister which we presented in the first case (Fig. 1) was diagnosed with FAP. Her mother was also diagnosed with CRC and died at age 55. This case had mutation at codon 849 of exon 15, a frameshift mutation with small deletion of two nucleotides (TA) (Fig. 6).This variant was considered as a family mutation since it was detected in his sister as well. Further clinical data were not available on this case.

**Case 4**

In this case, we present a 24-year-old male with severe polyposis at colon and germline muta-tion at codon 1309 (c. 3927_3931delAAAGA) (Fig. 7) of APC gene. According to the pedigree, family history of colorectal cancer is detected in every generation (Fig. 8). Further clinical data about the status of malignancy were missing in this case.

**Fig. 5:**
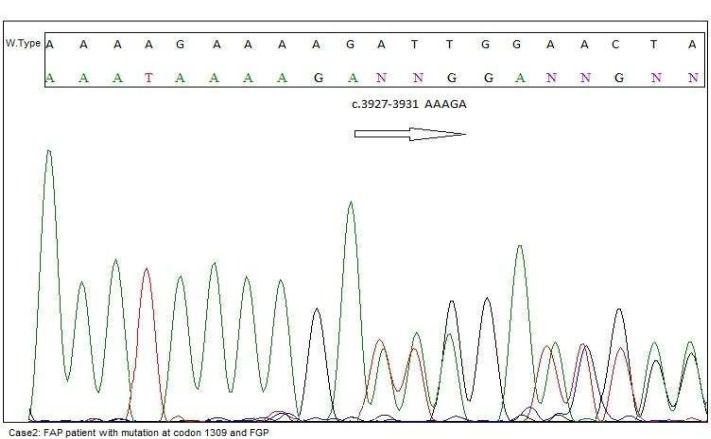
Screening of the APC gene in this case revealed a frameshift Mutation at codon 1309 (c.3927_3931del AAAGA p.Glu1309fs). Mutation at this codon associated with extracolonic manifestation like fundic gland polyps (FGP

**Fig. 6:**
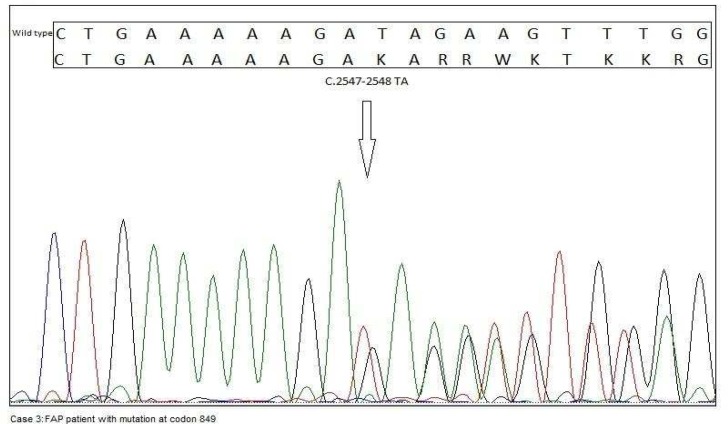
This case was identified with mutation at codon 849 (c.2547_2548delTA p.Asp849fsX62) in APC gene, a frameshift mutation with small deletion of two nucleotides (TA).

**Fig. 7:**
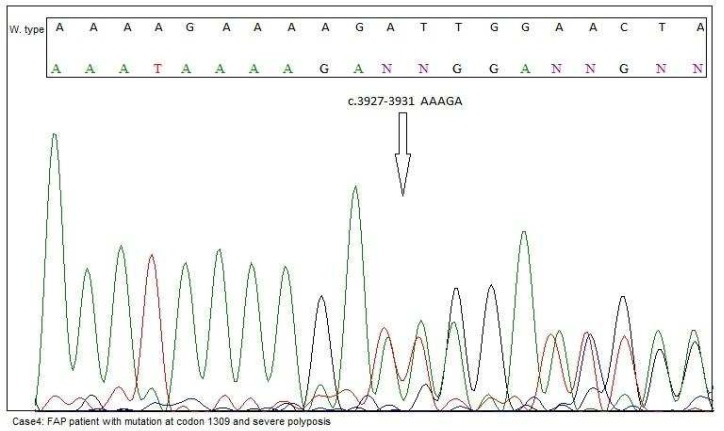
Mutation at codon 1309 (c.3927_3931del AAAGA p.Glu1309fs) with profuse polyposis was detected in this case

**Fig. 8:**
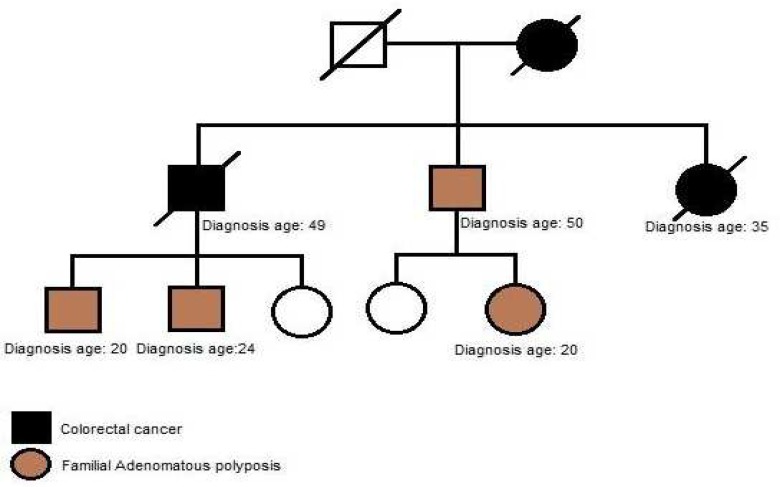
According to the pedigree, family history of colorectal cancer and FAP was detected in every generation. Patient diagnose with FAP at age 24.

## Discussion

In this case series which is the first study of phenotype and genotype correlation in Iranian FAP patients, we reported four FAP cases harboring frameshift mutation in exon 15 of APC gene. We found two siblings with germline mutation at codon 849 (c.2547_2548delTA p.Asp849fsX62). The other two patients had frameshift mutation at codon 1309 (c.3927_3931delAAAGA) which was reported in many previous studies (-). So far, two articles reported the occurrence of FAP in Iranian population. The previous study by Shahmoradgoli et al*.* on ten unrelated Iranian FAP patients identified 5 mutations at exon 15 of APC gene. They reported three nonsense and two missense mutations. In consistence with this study, one of the cases had mutation at codon 1309 but they did not report any clinical data of the case (18).

In a second study in 2007, Amini reported a 17 years old case with FAP. The patient was previously diagnosed with FAP at age 5 with several tubulovillous adenoma in the colon and underwent partial colectomy when he was 6 years old. Total colectomy and followed anastomosis was performed at age 11. The patient had several polyps in stomach, duodenum and jejunum (19). In this study, he was not screened for the mutation status in APC gene. Two out of four patients in our study had mutation at codon 1309 (5bp deletion, c.3927_3931 delAAAGA). Kim et al. in 2005 reported that codon 1309 (5bp deletion, c.3927_3931 delAAAGA) was the most common variant in their study and was detected in 6 patients (10%) (14). They also detected a frameshift mutation at codon 849 (c.2547_2548delTA p.Asp849fsX62) which was also found in two siblings in our study. The other study by Torrezan et al. (15) revealed that two out of 23 cases carry mutation at codon 1309 (delAAAGA). They observed severe polyposis (>1000) in two cases and mean age at diagnosis were 35 and 18 years respectively. Duodenal, gastric polyps and osteomas were detected in both cases. The low frequency of 1309 mutations in their study and lack of 1061 mutations imply the heterogeneity of Brazilian population. In our patient with the latter mutation, we found few hyperplastic polyps (5.5 mm) without any dysplasia or malignancies in fundus and antrum. In this case we also detected multiple adenomatous polyps with low grade dysplasia in duodenum. In a study of 24 Chilean FAP patients by De la Fuente et al. the most frequent mutation was c.3927_3931delAAAGA, found in 3 of 21 families (14%) (16). Kanter-Smoler et al. in 2008 reported that 10 out of 95 FAP cases carried mutation at codon 1309 (c.3927_3931del) (20). Similar to De la Fuente et al. finding, this aberrant alteration was the most frequent variation found in their study. In 2007 Aretz et al. reported denovo mutation at codon 1309 in two FAP cases (21). The other study by Plawski et al. revealed that c.3927_3931-delAAAGA mutation occurred in 10.3% of unrelated Polish FAP families (17). This was the most common mutation found in Polish FAP patients in their study. Another study by Rivera et al. in 2010 revealed that in 136 Spanish FAP families mutation at codon 1309 presented in 12/101 (11.9%) of the population and the average age of onset was 21 years (22). Extra colonic manifestations such as upper gastrointestinal polyps (UGP) were detected in one of our cases with mutation at codon 1309. This is consistent with the study of Rivera et al. (22) who reported 5 patients with the latter mutation and UGP. In the study of Torrezan et al. (15) two cases with 1309 mutations had gastric polyps and Kanter-Smoler et al. (20) also detected 5 patients with fundic gland polyps. In contrast to our study, De la Fuente et al. (16) and Plawski et al. (17) did not report any UGP related to FAP patients harboring mutation at codon 1309.

Congenital hypertrophy of retinal pigment epithelium (CHRPE) was not detected in our patients. Our finding is concordant with Kanter-Smoler et al.’s (20) and De la Fuente et al.’s (16) findings but not with Kim et al. (14) and Rivera et al. (22). Three patients with mutation at codon 1309 presented CHRPE in the study of Kim et al. in 2005 (14). Osteomas or dental abnormality was not detected in our study. This is in contrast with other studies (20, 14). Similar to Torrezan et al.’s (15) and De la Fuente et al.’s (16) studies, we detected duodenal polyp in one patient with mutation at codon 1309. Since dense polyposis is one of the main features of patients with mutation at codon 1309, in one patient, we detected a mild phenotype. Our finding is in contrast with the results of other studies (10, 15, 20) but in consistence with Aretz et al. (22) who detected two patients with attenuated form of the disease. This is interesting that in their study both patients were denovo cases but we did not observe the similar condition. In 1999, Won et al. determined the mutation status of APC gene in 62 unrelated Korean FAP patients (23). They found one patient with mutation at codon 849(c.2547-_2548delTA p.Asp849fsX62). Similar to our findings this patient had severe polyposis and presented desmoid tumor which is also consistent with the study of Kim et al. (14). Upper gastrointestinal polyp was also detected by Kim et al. which was not observed in our patient. In conclusion, this study is the first report of phenotype and genotype correlation in Iranian FAP patients. In our study in contrast to other studies, we revealed that one patient with mutation at codon 1309 had attenuated form of phenotype. To get a precise effect of frameshift mutation in FAP patients, we would need to enlarge our sample size and further screening of this pattern of mutation in affected cases would help our understanding of genotype and phenotype correlation in Iranian FAP patients.
